# Age-related directional asymmetry in the rod-and-frame test

**DOI:** 10.3389/fnagi.2026.1729404

**Published:** 2026-03-05

**Authors:** Michał Adamski, Miroslaw Latka, Anna Latka, Sławomir Wudarczyk, Tadeusz Sebzda, Bruce J. West

**Affiliations:** 1 Department of Biomedical Engineering, Wroclaw University of Science and Technology, Wroclaw, Poland; 2 Department of Neurology, St. Hedwigs Regional Specialist Hospital, Institute of Medical Science, University of Opole, Opole, Poland; 3 Department of Fundamentals of Machine Design and Mechatronics Systems, Wroclaw University of Science and Technology, Wroclaw, Poland; 4 Department of Physiology and Pathophysiology, Medical University of Wroclaw, Wroclaw, Poland; 5 Center for Nonlinear Studies, University of North Texas, Denton, TX, United States

**Keywords:** aging, directional asymmetry, field dependence, pseudoneglect, rod-and-frame test

## Abstract

**Introduction:**

In the classic Rod-and-Frame Test (RFT), participants align a pivoted rod with the vertical while viewing a tilted, coaxially mounted frame. In doing so, they can use the edge of the frame or its imaginary diagonal as visual cues. The error in this simple perceptual task has long been used to distinguish field-dependent from field-independent cognitive styles. Recent findings indicate that aging increases field dependence. Here, we test whether the shape of the RFT error curve as a function of frame tilt changes with age.

**Methods:**

Thirty-nine young adults (range: 19–27 years) and 50 older adults (range: 57–81 years) completed a virtual-reality version of the RFT. Each participant determined the vertical for nineteen frame tilt angles ranging from −45° to 45° in 5° increments.

**Results:**

At five clockwise tilts (10°–30°) and one counterclockwise tilt (−15°), older adults showed higher median errors than young adults, with the largest difference at 15° (7.5° vs. 2°). The asymmetry index of the RFT error curve was symmetrically distributed around zero in young adults but significantly positive in older adults, particularly those older than 68 years. Analysis of visual cue selection revealed that, for clockwise tilts, young adults flexibly switched between edge-based and diagonal-based positioning strategies depending on frame tilt magnitude, whereas older adults predominantly relied on edge-based cues. For counterclockwise tilts, differences in cue use between cohorts were minor. The asymmetry index was independent of overall performance, showing no correlation with the mean RFT error in either group.

**Conclusions:**

We propose that aging affects RFT performance through two dissociable mechanisms. A general decline in multisensory integration increases overall errors symmetrically across tilt angles. The clockwise-specific asymmetry, by contrast, may reflect age-related changes in lateralized visuospatial attention—specifically, the well-documented rightward attentional shift that accompanies healthy aging—which differentially affects the weighting of visual cues for clockwise vs. counterclockwise frame orientations.

## 1 Introduction

Aging is characterized by progressive molecular, cellular, and physiological changes that manifest as declines in muscle strength, sensory abilities, and cognitive performance (Guo et al., 2022; L'opez-Ot'in et al., 2023; Li et al., 2024). Intersubject variability in aging trajectories arises from differences in genetic background, lifestyle factors, and environmental exposures. Biomarkers of aging are instrumental in monitoring the aging process and assessing the efficacy of interventions. A recent expert consensus (Perri et al., 2025) identified 14 potential biomarkers spanning *physiological* (insulin-like growth factor 1, growth differentiation factor 15), *inflammatory* (high-sensitivity C-reactive protein, interleukin-6), *functional* (muscle mass, muscle strength, hand grip strength, Timed-Up-and-Go, gait speed, standing balance test, frailty index, cognitive health, blood pressure), and *epigenetic* (DNA methylation/epigenetic clocks) domains. In the proposed list, all categories are fairly specific except for cognitive health, underscoring the need for behavioral measures that capture age-related cognitive decline.

As early as 1948, Witkin and Asch performed several experiments to examine how visual surroundings affect human perception of the vertical (Asch and Witkin, 1948a,b; Witkin and Asch, 1948a,b). In one study, they discovered that a coaxially mounted, tilted frame causes humans to position a pivoted rod past vertical in the direction of the frame (Witkin and Asch, 1948b)—an experiment now called the Rod-and-Frame Test (RFT).

Later studies showed that individuals differ systematically in their RFT errors, leading Witkin to formulate the Field Dependence-Independence (FDI) construct. In the now-classic text “Personality through Perception: An Experimental and Clinical Study,” Witkin argued that the ability to perceive visual elements independently of their surrounding context, as measured by the RFT, reflects broader cognitive, social, and personality characteristics (Witkin et al., 1954).

Recent interest in the RFT has been renewed by findings that aging consistently shifts cognitive style from field-independent to field-dependent (Chan and Yan, 2018). Older adults increasingly rely on their visual environment to guide their behavior and performance.

Accurate perception of the vertical requires integrating visual, vestibular, and proprioceptive cues (Böhmer and Mast, 1999; Clarke et al., 2003; Anastasopoulos et al., 1999; Trousselard et al., 2003; Barra et al., 2010; Pavlou et al., 2003; Hansson et al., 2010; Dockheer et al., 2018), making the RFT a valuable paradigm for studying how this multisensory integration changes with aging.

The error in determining the vertical in the RFT is a sinusoidal function of frame tilt, with the maximum deviation toward the tilted frame occurring between 18° and 28° (Witkin and Asch, 1948b; Bagust, 2005; Zoccolotti et al., 1992). Therefore, many studies investigating the effects of aging on the RFT have employed only one tilt angle within this range, together with its opposite (De Lisi and Smith, 1979; Bagust et al., 2013; Razzak, 2013; Willey and Liu, 2022). Here, we use a larger set of tilt angles (from −45° to 45°) to test whether not only the maximal error but also the overall shape of the RFT error curve changes with aging, reflecting different strategies used by young and older adults to determine the vertical. In particular, we aim to establish whether the symmetry of the error curve is affected by aging.

Interestingly, age-related changes in visuospatial attention may also influence RFT performance. Young adults exhibit pseudoneglect—a leftward attentional bias attributed to right-hemisphere dominance for spatial processing—which typically attenuates or reverses with aging, producing a rightward shift in spatial attention (Jewell and McCourt, 2000; Benwell et al., 2014). Whether this lateralized attentional shift produces asymmetric RFT errors—with differential performance on clockwise vs. counterclockwise frame tilts—has not been investigated.

In the RFT, participants estimating the vertical may rely on the frame's edge or its imaginary diagonal as visual cues. Relying on either cue inevitably produces an error (the RFT illusion). In our recent work, we showed that young adults who can flexibly use *both* cues—the edge at small tilts and the diagonal at large tilts—perform significantly more accurately at angles where errors typically peak (Adamski and Latka, 2025). The second goal of this study is to test whether older adults exhibit similar strategic flexibility in visual cue selection. To this end, we employ the bias function (Adamski and Latka, 2025), which quantifies whether participants position the rod toward the frame's edge or its diagonal at each tilt angle.

## 2 Subjects and methods

### 2.1 Subjects

The research protocol was approved by the Opole Ethics Committee and adhered to the principles outlined in the Declaration of Helsinki. All participants voluntarily took part in the study and provided written informed consent. All participants reported normal or corrected-to-normal vision. We recruited 39 healthy young adults (HY) (17 men and 22 women; age: 22 ± 1 years, range 19–27 years) from the student population of Wroclaw University of Science and Technology. All young participants reported no history of neurological disorders. The cohort of 50 healthy older adults (HO) (22 men and 28 women; age: 68 ± 6 years, range 57–81), all physically and mentally active, was recruited from several sources: 24 participants from a seniors' club in Marklowice (a suburban mining community), 18 from the University of the Third Age in Legnica, and 8 residents of local retirement homes. All older adults were familiar with using personal computers and underwent a comprehensive neurological assessment, including the Standardized Mini-Mental State Examination (SMMSE). The SMMSE scores ranged from 26 to 30, with a mean score of 28 ± 2. All participants self-identified as White and were native Polish speakers who read from left to right. Both cohorts had comparable educational backgrounds beyond high school. Exclusion criteria during recruitment included evidence of mild cognitive impairment and diagnosed neurological disorders (e.g., Parkinson's disease). One participant with an SMMSE score of 22 and one participant presenting parkinsonian symptoms were excluded from the study. During testing, participants were instructed to stop if they experienced dizziness, fatigue, or VR sickness symptoms; no participants discontinued for these reasons.

### 2.2 Software and study protocol

Supplementary Figure S1 shows the virtual reality test room. The scene was implemented in Unity 3D and displayed using an Oculus Rift headset. This headset has a hardware interpupillary distance (IPD) adjustment mechanism. IPD was adjusted for each participant at the start of the session. For each eye, the headset provides an 80-degree horizontal by 90-degree vertical field of view, with a resolution of 1080x1200 pixels. Built-in anti-aliasing was employed to smooth jagged edges. The graphics engine's unit of length corresponds to approximately 1 m. The subject looks at a rod that is 1.3 m long from a distance of 5 m. The width and height of the cuboid room are equal to 1.8 m. The scene is illuminated by a spotlight placed behind the subject, who attempts to align the rod with the vertical for 19 randomly presented angles of frame tilt θ (from −45° to 45° in steps of 5° with respect to the vertical).

In our convention, a counterclockwise frame rotation corresponds to a negative θ, while a clockwise rotation corresponds to a positive θ. Throughout this paper, we use “positive tilts” and “clockwise tilts” interchangeably (both refer to θ>0), and similarly for “negative” and “counterclockwise” tilts (θ < 0).

The rod always started 5° closer to the vertical than the tilted frame, except at θ = 0°, where it was initially rotated 5° clockwise. The subject adjusts the rod angle using a wireless computer mouse wheel and verbally confirms task completion. The sensitivity of the mouse wheel is equal to 0.5°, which corresponds to the value used by Bagust (2005). The mouse wheel was chosen as the input device because older adults, many of whom were unfamiliar with VR controllers, found it intuitive to use. The observed errors were substantially larger than the input resolution, suggesting that the input discretization did not limit the measurement's precision.

The participants were seated with their back against the backrest of the chair and their feet flat on the floor. After the goggles were comfortably fitted, they familiarized themselves with the virtual reality environment using a variant of the Virtual Reality Rod and Frame Test (VRRFT) software that explained the rod's positioning. After approximately 5 min of familiarization, the actual test began with the frame always visible. For a given frame tilt, once the participant completed positioning the rod, the frame smoothly returned to the vertical position and remained there for 2 s.

Before the experiment began, participants received verbal instructions in their native language. They were asked to align a virtual rod with gravity. The instructions were as follows:

“*Your task is to rotate the rod so that it aligns with the vertical that is, the direction gravity pulls straight downward. Imagine the rod is hanging freely from its upper end, like a plumb line. Try to ignore the tilted frame. Focus only on what you believe is the direction of gravity in the real world.”*

The VRRFT session typically lasted 15–30 min per participant, depending on their progress. There was no time limit.

### 2.3 Data analysis

#### 2.3.1 RFT error asymmetry index

Let ${\left\{e\left(\theta_{k}\right)\right\}}_{k=1}^{N_{k}}$ be the set of errors a subject makes when determining the vertical for *N*_*k*_ frame tilts θ_*k*_. We denote by *E*_+_ the sum of the absolute value of the alignment error for clockwise (positive) frame tilt angles:


$$\begin{array}{l} E_{+}=\underset{\theta_{k}>0}{\sum }|e\left(\theta_{k}\right)|. \end{array}$$ (1)

*E*_−_ is the corresponding sum for the counterclockwise (negative) tilt angles:


$$\begin{array}{l} E_{-}=\underset{\theta_{k}<0}{\sum }|e\left(\theta_{k}\right)|. \end{array}$$ (2)

We quantify the asymmetry of the subject's error curve using the following index:


$$\begin{array}{l} \alpha_{e}=\frac{E_{+}-E_{-}}{E_{+}+E_{-}}. \end{array}$$ (3)

Due to the geometric symmetry of the frame, trials at ±45° (where the frame's diagonals are vertically aligned) were excluded from asymmetry calculations, as were trials at 0° (where the frame's edge is vertical and no tilt-induced bias can occur). The asymmetry index α_*e*_ ranges from −1 to 1, with positive values indicating that the magnitude of the errors is greater for positive (clockwise) frame tilts.

#### 2.3.2 Error bias

**Figure 1:**
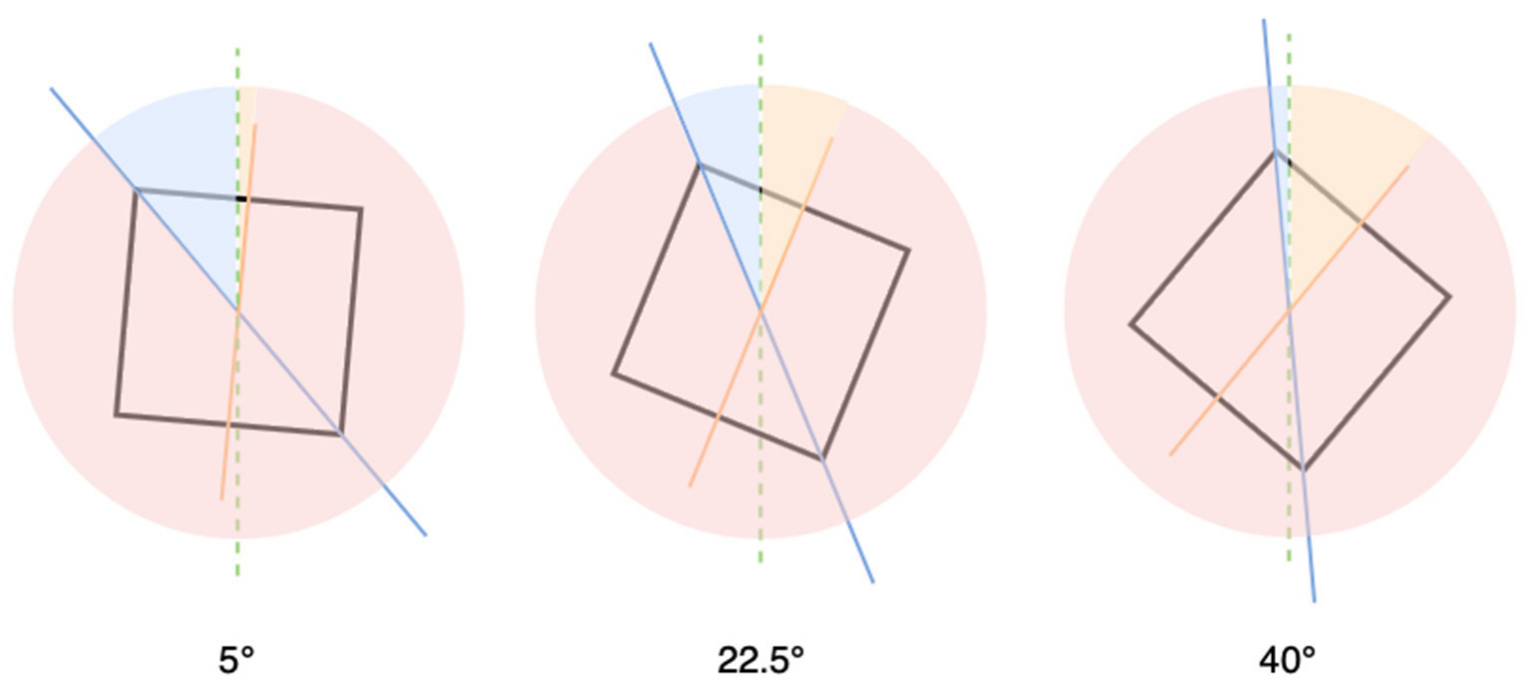
During the Rod and Frame Test (RFT), a subject attempts to align the rod with the vertical. The edges of the frame, represented by the orange line, and the imaginary diagonals (blue) can act as visual cues. For simplicity, we display only one diagonal. With small frame tilts (e.g., 5°), subjects tend to position the rod toward the rotated frame, resulting in an error within the yellow-shaded arc. In this instance, the sign of the error matches the sign of the frame tilt (direct frame effect). The alternative strategy—aligning with the diagonal—would lead to a much larger error, as shown by the blue-shaded arc. Here, the error's sign is opposite to the frame tilt (indirect frame effect). For a frame tilt of 22.5°, either strategy would produce a comparable error. However, with a large frame tilt (e.g., 40°), aligning with the diagonal may be advantageous, as demonstrated by the relative widths of the arcs for the direct and indirect effects.

The edges of the frame and its imaginary diagonals can serve as visual cues that subjects may use to estimate the vertical. For small frame tilts, participants tend to align the rod toward the rotated frame (Figure 1). In this case, the sign of the error *e*(θ) matches the sign of the tilt angle θ. Abdul Razzak and Bagust (2024) describe this strategy as the *direct* effect. An alternative strategy—aligning with the diagonal—would produce a substantially larger error, but becomes advantageous for large frame tilts. Under this condition, θ and *e*(θ) have opposite signs. Following the terminology of Abdul Razzak and Bagust (2024), we refer to this as the *indirect* effect. Importantly, reliance on either of these visual cues inevitably results in the RFT error.

Let ${\left\{\left(\theta_{i},e_{i}\right)\right\}}_{i=1}^{N}$ be the set of tests, where θ_*i*_ is the tilt angle of the frame for the test *i*, and *e*_*i*_ = *e*(θ_*i*_) is the corresponding error. The set $\left\{E\right\}={\left\{e_{i}\right\}}_{i=1}^{N}$ comprises the errors made by the cohort members for a given frame tilt or the errors made by a subject over a range of frame tilts. *E*^(direct)^ and *E*^(indirect)^ are the number of trials for which direct and indirect effects are observed:


$$\begin{array}{l} E^{\left(\text{direct}\right)}=|\left\{i:\text{sign}\left(e_{i}\right)=\text{sign}\left(\theta_{i}\right)\right\}|, \end{array}$$ (4)

$$\begin{array}{l} E^{\left(\text{indirect}\right)}=|\left\{i:\text{sign}\left(e_{i}\right)=-\text{sign}\left(\theta_{i}\right)\right\}|. \end{array}$$ (5)

In the above equations, |·| denotes the cardinality of the set. We then define the bias function as


$$\begin{array}{l} b=\frac{E^{\left(\text{direct}\right)}-E^{\left(\text{indirect}\right)}}{N}, \end{array}$$ (6)

where *N* is the total number of trials.

The bias *b* can vary between −1 and 1. When only a direct effect is observed, the function takes the value of 1. In contrast, a value of −1 indicates an indirect effect.

For both cohorts, we calculate the bias for each angle of frame tilt separately. In addition, we divide the frame tilt range into intervals based on observed discontinuities in the bias function, allowing data-driven characterization of cue usage patterns across different frame tilts.

### 2.4 Statistical analysis

Normality of data was assessed using the Shapiro–Wilk test. Because most distributions were non-Gaussian, nonparametric statistics were used for these variables. Continuous variables with non-normal distributions are reported as median with interquartile range [Q1, Q3], where Q1 and Q3 represent the first and third quartiles, respectively. Between-group comparisons for these variables were performed using the Mann–Whitney *U* test, and one-sample tests for deviation from zero used the Wilcoxon signed-rank test. The asymmetry index (α_*e*_) was approximately normally distributed in both cohorts (see Results), and is therefore reported as mean ± standard deviation; between-group comparison used the independent-samples *t*-test, and deviation from zero was tested with one-sided one-sample *t*-tests. For multiple comparisons, the Bonferroni correction was applied. Statistical significance was set at α = 0.05. Confidence intervals for the group-averaged RFT error curves were estimated using a nonparametric percentile bootstrap based on 100,000 resamples.

To examine whether directional asymmetry increased with advancing age within the older cohort, we compared participants above and below the median age of 68 years. This threshold coincides with the age at which population-based studies have reported accelerated decline in both cognitive and motor function (van der Willik et al., 2021).

Sample sizes were determined by practical recruitment constraints. As a post-hoc sensitivity analysis, we estimated the effect sizes detectable with 80% power at α = 0.05 (two-tailed). For the between-group comparison (*n*_1_ = 39, *n*_2_ = 50), the study was sensitive to effects of *d*≥0.60. For the one-sample test in older adults (*n* = 50), the study was sensitive to effects of *d*≥0.40. For the comparison of older adult subgroups split by median age (*n*_1_ = 24, *n*_2_ = 26), the study was sensitive to effects of *d*≥0.79.

All analyses were performed using Wolfram Mathematica 14.2.

## 3 Results

### 3.1 RFT errors

**Figure 2:**
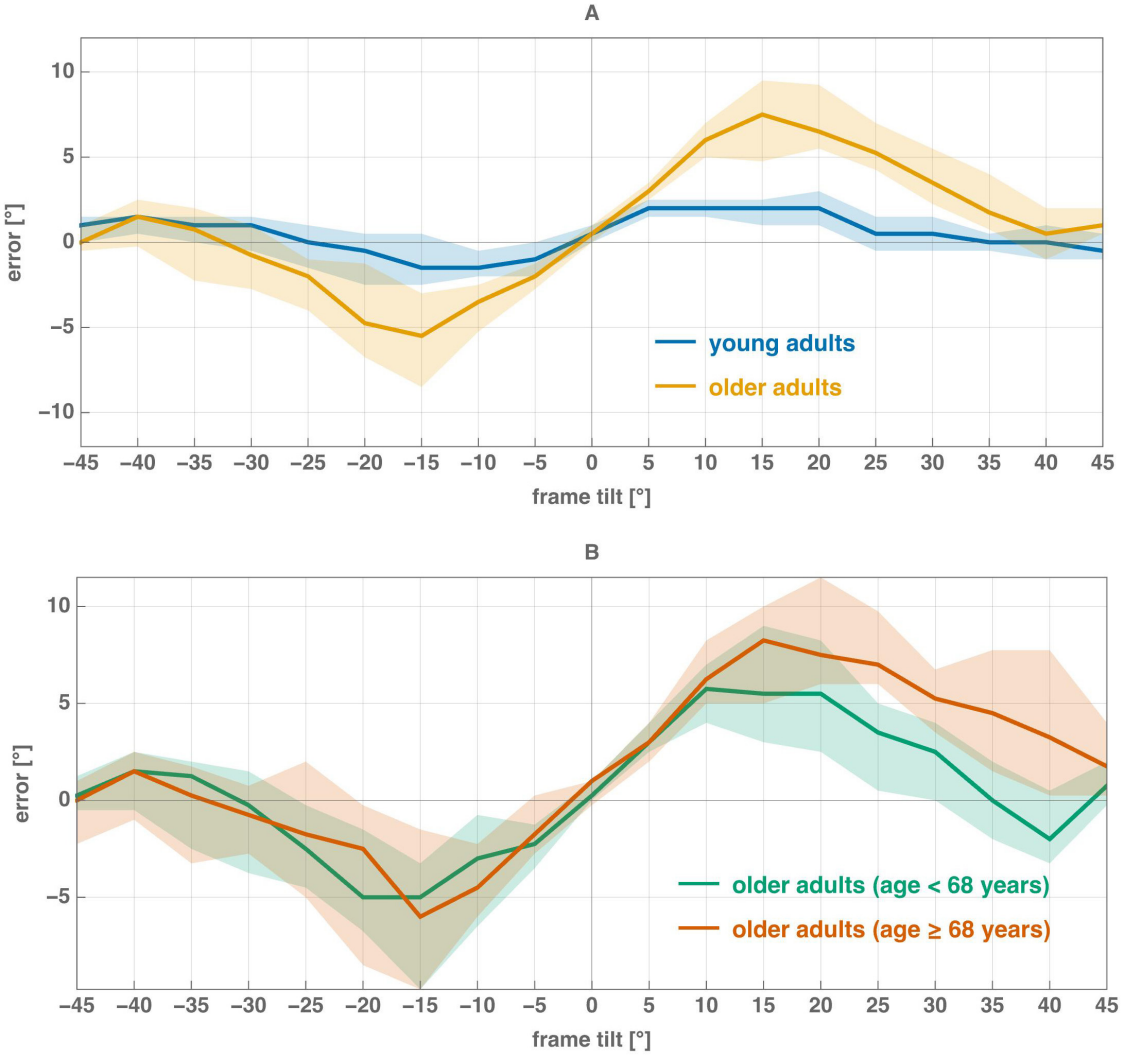
Median error *e*(θ) in determining the vertical is plotted as a function of frame tilt θ: **(A)** young and older adults, **(B)** older adults split into two subgroups relative to the median age of the cohort (68 years). Shading indicates the 95% confidence interval, estimated using the bootstrap method.

The median error *e*(θ) for all frame tilt angles is shown in Figure 2A for both cohorts. Older adults showed significantly higher errors than young adults at positive tilts from 10° to 30° and at −15° (Mann–Whitney *U* test with Bonferroni correction, all *p* < 0.05/19), with the largest difference at 15° (HO: 7.5°, HY: 2.0°). No significant differences emerged at other angles.

We observed no significant differences in alignment error between males and females (Supplementary Figure S2 and Supplementary Tables S1, S2).

In Figure 2, there is a small positive median alignment error for the vertical frame (θ = 0°). This error was statistically greater than zero for the older adult cohort as a whole (median = 0.50°, *p* = 0.01) and for the subgroup younger than 68 years (median = 0.25°, *p* = 0.02), but not for older adults aged 68 years or above (*p* = 0.10) or for young adults (*p* = 0.06); see Table 1. In all cases, the median error was comparable to or smaller than the mouse wheel resolution (0.5°).

**Table 1:** Median alignment error for the non-tilted frame (θ = 0°).

**Group**	***Mean*±*SD***	***Median*[*IQR*]**	***p*-value**
Young	0.37 ± 1.39	0.50 [−0.50, 1.00]	0.06
Older < 68	0.56 ± 1.16	0.25 [0.00, 1.25]	**0.02**
Older ≥68	0.42 ± 1.53	1.00 [−0.50, 1.00]	0.10
Older	0.49 ± 1.36	0.50 [−0.38, 1.00]	**0.01**

Values are reported as mean ± SD and median [IQR]. *p*-values correspond to one-sample Wilcoxon signed-rank tests assessing whether the alignment error differs from zero within each group. Bolded *p*-values indicate values below the 0.05 significance level.

### 3.2 RFT error curve asymmetry

**Figure 3:**
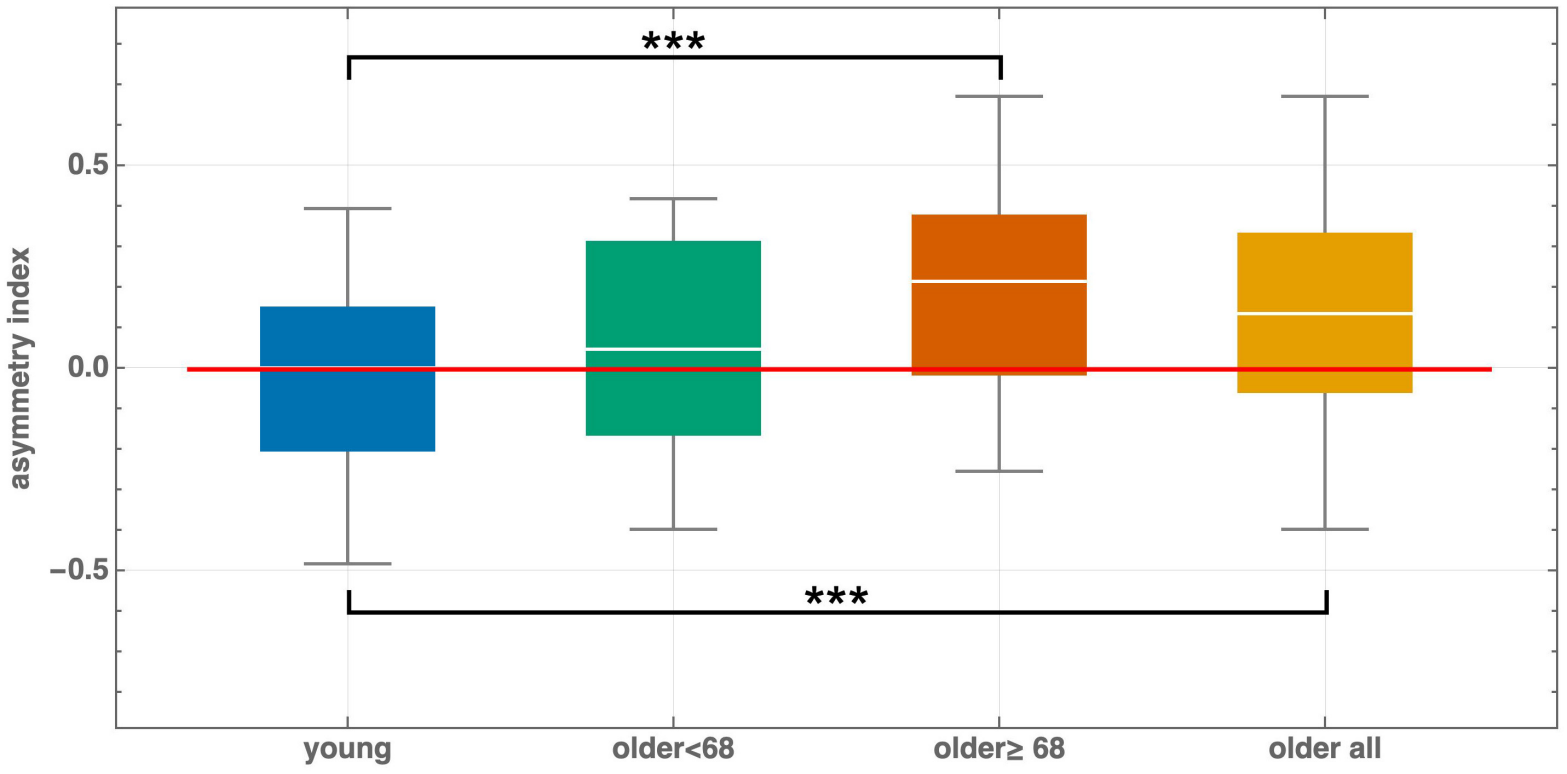
Asymmetry index for young and older adults. The index is also shown for participants younger or older than 68 years (the median age of the older adult cohort). ***p-value < 0.001.

The group-averaged error curve of young adults was relatively symmetric, in contrast to that of older adults (Figure 2A). Furthermore, comparison of older adult subgroups divided by the median age revealed that asymmetry increased with advancing age (Figure 2B). To quantify these patterns, we calculated the asymmetry index α_*e*_ for each participant (Figure 3).

In both cohorts, α_*e*_ was approximately normally distributed and showed no outliers (IQR rule). Shapiro–Wilk tests were non-significant for young (*W* = 0.977, *p* = 0.60) and older adults (*W* = 0.979, *p* = 0.51). Homogeneity of variance was supported by Levene's test (*F*(1, 87) = 0.77, *p* = 0.38).

For young adults, α_*e*_ = −0.02 ± 0.23. A one-sample *t*-test indicated that the mean did not differ significantly from zero (*t*(38) = −0.63, *p* = 0.53).

For older adults, α_*e*_ = 0.12 ± 0.26 was significantly greater than zero (*t*(49) = 3.43, *p* = 0.0006), corresponding to a medium effect size (Cohen's *d* = 0.49).

The between-cohort difference was statistically significant, with older adults showing larger values than young adults (*t*(87) = −2.82, *p* = 0.006), corresponding to a medium effect size (Cohen's *d* = 0.60).

There was no significant correlation between age and the asymmetry coefficient within the older adult cohort (Pearson's *r* = 0.15, *p* = 0.29). Participants older than the median age of 68 years exhibited asymmetry index values approximately four times greater than those aged 68 years or younger (0.21 vs. 0.05; *t*(48) = 2.25, *p* = 0.03, Cohen's *d* = 0.63). Although statistically significant, this comparison did not reach the sensitivity threshold for 80% power (*d*≥0.79). The asymmetry index for this subcohort was statistically higher than that of young subjects (*t*(67) = 3.74, *p* = 0.0003).

**Figure 4:**
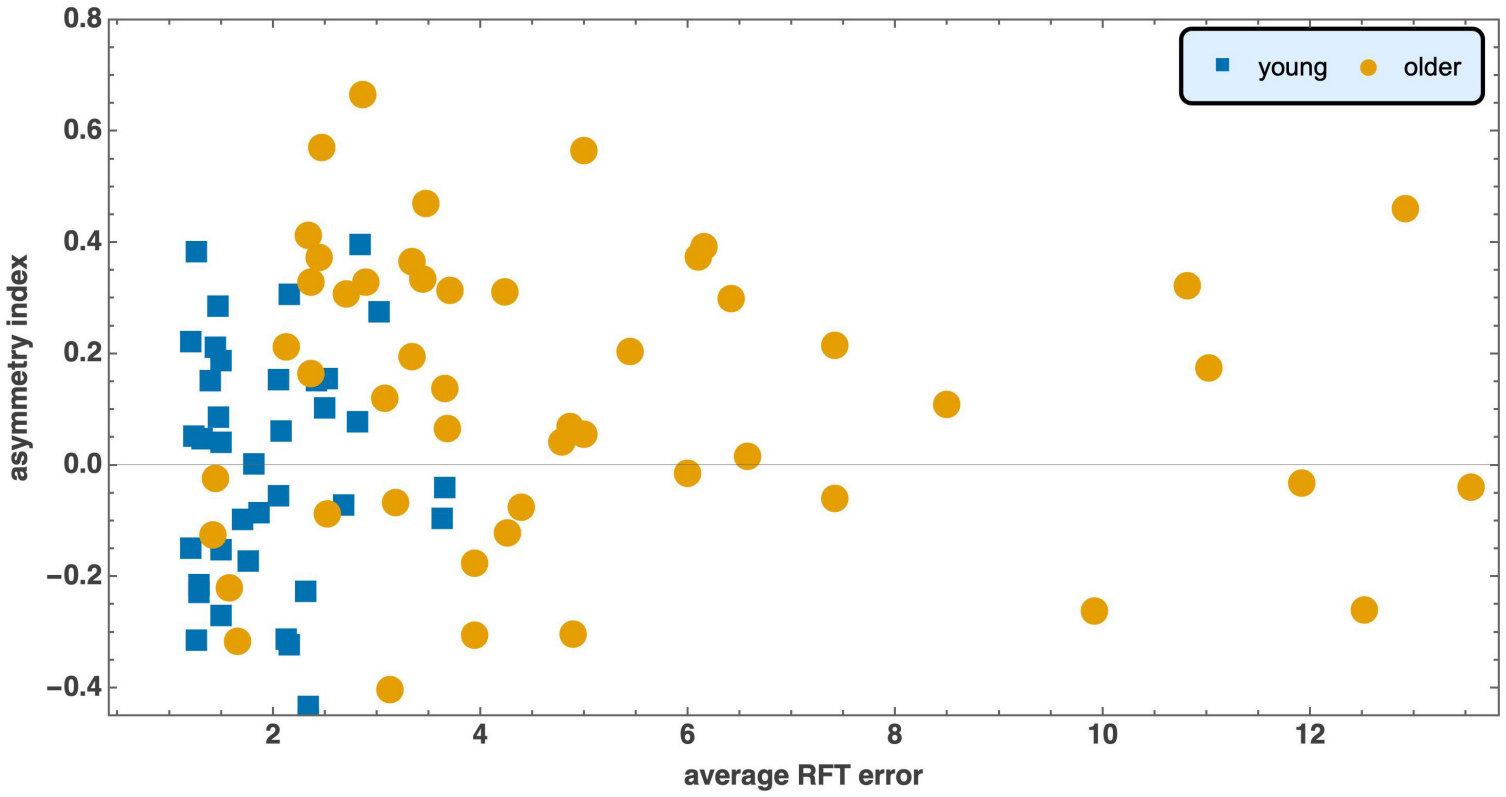
Asymmetry index vs. average RFT error is plotted for young and older participants.

Finally, the scatter of points in Figure 4 indicates no association between asymmetry and overall RFT performance in either cohort. Consistent with this observation, Spearman's correlation between the absolute asymmetry index and the mean absolute error was non-significant for both young adults (ρ = −0.09, *p* = 0.56) and older adults (ρ = −0.19, *p* = 0.18).

### 3.3 Cohort differences in visual cue selection

To characterize how cue preference varies with frame tilt magnitude, we identified four intervals showing qualitatively distinct bias patterns (Figure 1). Group-averaged bias values for these intervals (Table 2) revealed three key findings.

**Table 2:** Group-averaged bias by frame tilt interval and age cohort.

**Frame tilt interval**	**Young (HY)**	**Older (HO)**
	***n* = 39**	***n* = 50**
[−45°, −25°]	−0.27	0.06
[−20°, −5°]	0.31	0.47
[5°, 20°]	0.69	0.80
[25°, 45°]	0.07	0.47

For both cohorts, bias was calculated for each frame tilt angle, then averaged across the four intervals shown. Bias ranges from −1 (diagonal-based positioning, or indirect effect) to +1 (edge-based positioning, or direct effect).

First, both cohorts exhibited pronounced directional asymmetry: bias at moderate negative tilts [−20°, −5°] (HY: 0.31, HO: 0.47) was approximately half that at corresponding positive tilts [5°, 20°] (HY: 0.69, HO: 0.80).

Second, young adults showed distinctive behavior at large positive tilts [25°, 45°], where mean bias dropped to near zero (0.07). In contrast, older adults maintained substantially higher bias (0.47), indicating persistent edge-based positioning.

Third, at large negative tilts [−45°, −25°], young adults showed negative mean bias (−0.27), while older adults' bias was near zero (0.06).

### 3.4 Individual variability in cue selection

**Figure 5:**
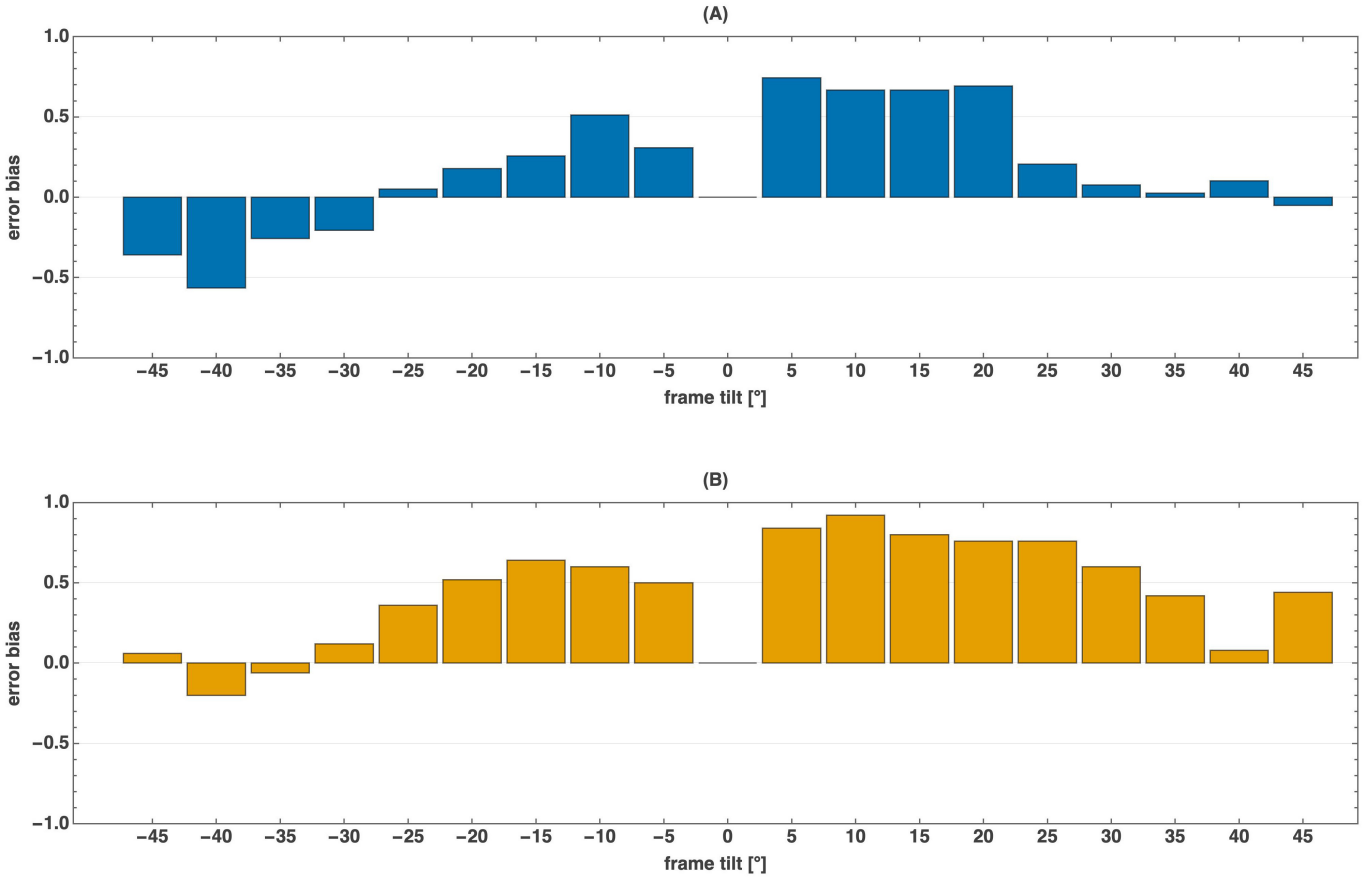
Error bias *b*(θ) for young **(A)** and older **(B)** adults as a function of frame tilt θ. Bias is high in the interval [5°, 20°] (0.69 for young, 0.80 for older adults), indicating that participants rotated the rod past the vertical in the direction of the frame tilt. For larger positive tilts ([25°, 45°]), older adults continued to rely on frame edges (bias = 0.47), whereas young adults often used diagonals (bias = 0.07). The bias function is asymmetric: in young adults, bias in [−20°, −5°] was about half of that in [5°, 20°] (0.31 vs. 0.69).

While group-averaged bias revealed systematic patterns, individual participants showed considerable variability in their cue selection strategies (Figure 5).

At moderate positive tilts [5°, 20°], median bias for both young and older adults was 1.00, indicating strong preference for the edge cue. The corresponding interquartile ranges were [0.50, 1.00] for young adults and [1.00, 1.00] for older adults.

For young adults at large positive tilts [25°, 45°], median bias dropped substantially to 0.20 (IQR: [−0.60, 0.60]), a statistically significant decrease from moderate tilts (Wilcoxon signed-rank test, *p* < 0.001). The large interquartile range indicates that some participants maintained edge-based positioning (direct effect) while others adaptively switched to diagonal-based positioning (indirect effect) depending on frame tilt magnitude.

In contrast, older adults showed more homogeneous bias distributions. At large positive tilts [25°, 45°], median bias for older adults decreased to 0.60 (IQR: [0.00, 1.00])—a smaller but still statistically significant change from moderate tilts (Wilcoxon signed-rank test, *p* < 0.001). Most individuals clustered in the positive range, and the reduced spread indicates that older adults relied on edge-based positioning more consistently regardless of tilt magnitude, even when this strategy became suboptimal.

Between-cohort comparison revealed that median bias at large positive tilts [25°, 45°] was significantly higher in older adults than in young adults (Mann–Whitney U test, *p* = 0.01). For the corresponding negative tilts [−45°, −25°], no significant difference emerged between cohorts after Bonferroni correction (*p* = 0.04>0.05/4).

## Discussion

Let us first examine the test results for clockwise frame rotations (θ>0). For both cohorts, small tilt angles elicited strong edge-based influences on rod positioning, as indicated by high bias values (Figure 1). Although *e*(θ) initially increased linearly with frame tilt in both groups, young adults exhibited a smaller slope and faster error saturation (Figure 2A), suggesting more efficient use of vestibular and proprioceptive cues for vertical alignment.

**Figure 6:**
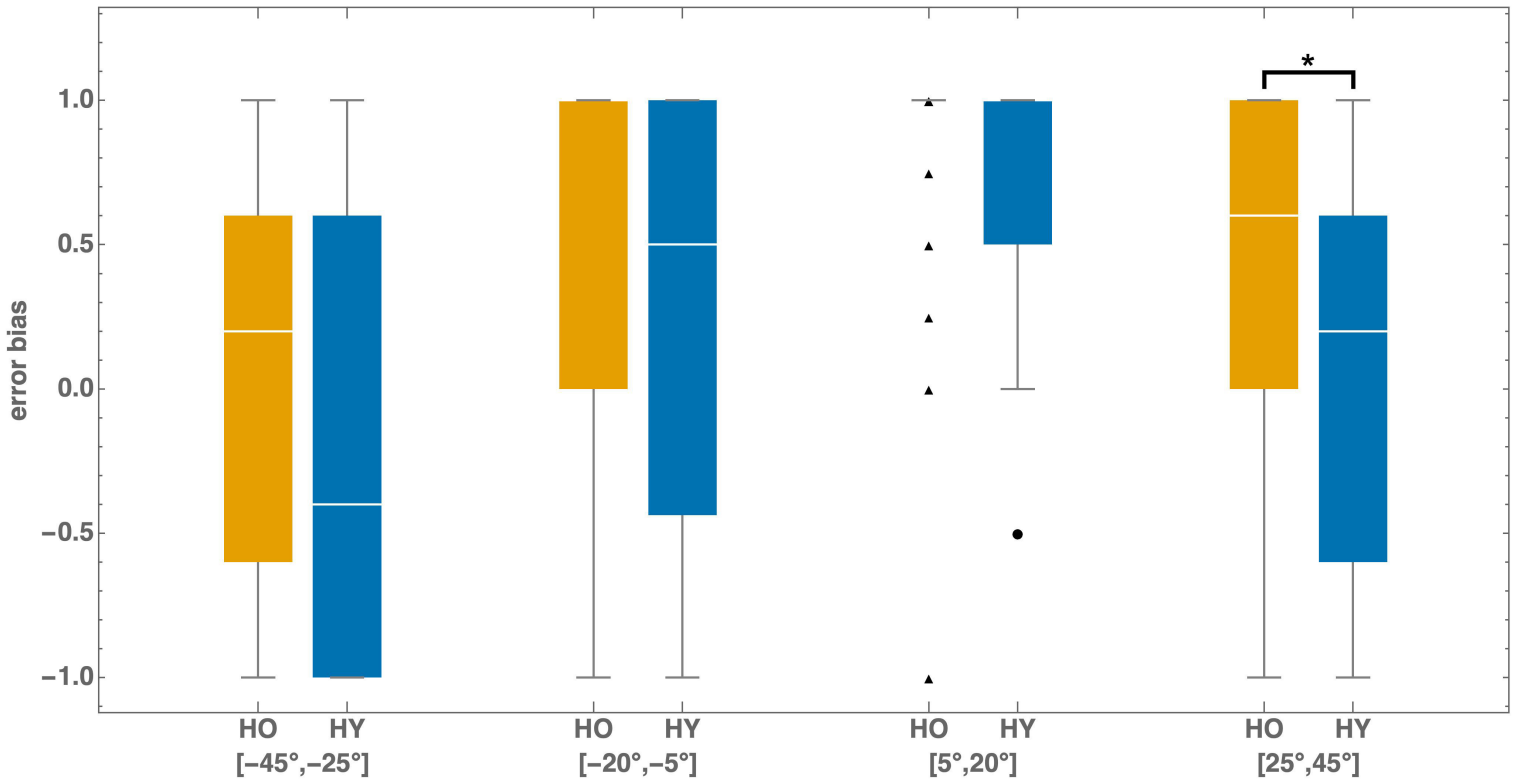
Distribution of error bias *b* for older (HO) and young (HY) adults. For each participant, bias was calculated in four frame tilt intervals: [−45°, −25°], [−20°, −5°], [5°, 20°], and [25°, 45°]. In the interval [5°, 20°], 41 of 50 older adults had *b* = 1, so the median equals 1 and only outliers are visible. For larger clockwise tilts ([25°, 45°]), many participants relied on the frame's diagonals rather than its edges, resulting in a significantly smaller median bias compared to [5°, 20°]. Note also the asymmetry: for both cohorts, the median bias in counterclockwise intervals was consistently smaller than in the corresponding clockwise intervals. *p-value < 0.05.

As θ approached 22.5°, sensory conflict intensified, making vertical estimation increasingly difficult, particularly for older adults. Some participants continued to rotate the rod toward the slanted frame even when its edges no longer provided useful information (Figure 6). In contrast, young adults showed a sharp drop in bias at 25°, which remained small and positive at larger tilts. Individual data (Figure 5) reveal that several young participants rotated the rod opposite to the frame's direction—an indirect effect previously described by Abdul Razzak and Bagust (2024)—consistent with the use of diagonal cues for alignment.

For positive angles ([25°, 45°]), most older adults persisted in edge-based positioning, as reflected by the high group-averaged bias (Figure 1) and the distribution of individual biases in this range (Figure 5). For HO subjects, the error in the determination of vertical becomes small at 40° (Figure 2A), indicating that only at very large frame tilts can they use diagonal cues to determine verticality.

The most striking finding of the present study was a pronounced *directional asymmetry* in age-related cohort differences. Specifically, for five clockwise frame tilts within the range [10°, 30°], older adults exhibited significantly larger alignment errors than young adults, whereas no comparable differences were observed for counterclockwise tilts (with the exception of −15°). Consistent with this pattern, the asymmetry index α_*e*_ was symmetrically distributed around zero in young adults but was significantly positive in older adults (0.12 ± 0.26, *p* = 0.0006, Cohen's *d* = 0.49), indicating a systematic bias toward larger errors at positive tilts. The between-cohort difference was also significant (*p* = 0.006, Cohen's *d* = 0.60). Moreover, this asymmetry appeared to increase with advancing age: participants older than 68 years exhibited α_*e*_ values approximately four times greater than those aged 68 years or younger (0.21 vs. 0.05; *p* = 0.03, Cohen's *d* = 0.63), although this subgroup comparison was underpowered and should be interpreted with caution. Taken together, these results indicate that aging is associated not merely with increased RFT error magnitude, but with a *direction-specific vulnerability*.

We propose that this directional asymmetry is consistent with the hypothesis of age-related alterations in lateralized spatial attention. Young adults reliably exhibit *pseudoneglect*—a leftward bias in visuospatial attention attributed to right-hemisphere dominance for spatial processing (Bowers and Heilman, 1980; Jewell and McCourt, 2000; Benwell et al., 2013). This bias is typically assessed using line bisection or landmark tasks, in which participants judge the midpoint of horizontal lines. Young adults systematically perceive the midpoint as being slightly to the left of the veridical center, consistent with enhanced attention to left hemispace (Benwell et al., 2013, 2014). With increasing age, this leftward bias typically attenuates or reverses, resulting in a rightward shift of spatial attention (Fujii et al., 1995; Schmitz and Peigneux, 2011; Benwell et al., 2014).

Crucially, the magnitude of this rightward attentional shift depends on task difficulty. Benwell et al. investigated age-related changes in visuospatial attention using a landmark task, in which participants judged whether a transection mark appeared closer to the left or right end of a horizontal line (Benwell et al., 2014). By varying line length, the authors manipulated perceptual demand: shorter lines are harder to discriminate and yield shallower psychometric functions. Young adults displayed the expected leftward bias (pseudoneglect) for long lines, with progressively attenuated bias for shorter lines. Elderly adults showed a qualitatively similar pattern but shifted rightward overall: they exhibited no systematic bias for long lines and, critically, a significant *rightward* bias for the shortest lines. Thus, the age-related rightward shift became most pronounced under the most demanding perceptual conditions—precisely when accurate performance required maximal attentional resources.

We suggest that the RFT presents an analogous challenge. At intermediate frame tilts, edge-based and diagonal-based visual cues provide conflicting estimates of verticality, creating perceptual ambiguity that must be resolved. Just as short lines in the landmark task tax attentional resources and unmask the rightward bias in elderly participants, intermediate frame tilts in the RFT may expose a similar vulnerability. Under these demanding conditions, the rightward attentional shift characteristic of aging may differentially affect the processing of clockwise vs. counterclockwise frame orientations.

Importantly, this interpretation is supported by independent experimental evidence. Razzak (Razzak, 2013) demonstrated that imposing time pressure on young healthy adults produces a clockwise-specific asymmetry closely resembling that observed in the present older cohort. Under time pressure, participants showed significantly increased alignment errors for clockwise tilts only, with no corresponding effect for counterclockwise tilts. Razzak attributed this effect to insufficient time to downweight misleading visual cues—a process ordinarily mediated by right-hemisphere mechanisms. Our findings suggest that aging could produce a *functionally equivalent deficit*, not due to temporal constraints but due to reduced right-hemisphere capacity. The convergence of this experimental manipulation with our age-related findings on the same directional asymmetry strengthens the interpretation that clockwise-specific vulnerability in the RFT reflects compromised right-hemisphere-mediated visual reweighting.

Notably, the observed asymmetry was significant only among participants older than the median age of 68 years. This finding is consistent with evidence that age-related rightward attentional shifts are nonlinear and accelerate with age. Benwell et al. reported that the rightward bias for short lines emerged specifically in their elderly sample (mean age 68 years), whereas young adults showed no systematic bias for these stimuli (Benwell et al., 2014). Our results closely mirror this pattern: the directional asymmetry in RFT performance becomes evident only beyond approximately 68 years of age, suggesting that lateralized attentional imbalance becomes behaviorally relevant only after crossing a critical threshold in the aging process (van der Willik et al., 2021).

A small but statistically significant positive bias was observed at θ = 0° in older adults (median = 0.50°, *p* = 0.01), indicating a slight clockwise offset in perceived verticality even without frame tilt. However, this baseline bias was much smaller than the directional asymmetry observed at intermediate frame tilts and falls well within the range of normal SVV variability reported in the literature (Barra et al., 2010).

What neural mechanisms might underlie this age-related rightward shift in spatial attention? Several accounts have been proposed. The most parsimonious explanation involves disproportionate aging of the right hemisphere (Brown and Jaffe, 1975), which would reduce right-hemisphere dominance for visuospatial processing and thereby shift the balance of spatial attention rightward. Consistent with this account, electrophysiological studies have demonstrated that right parieto-occipital responses to spatial stimuli present in younger adults are absent or markedly reduced in older adults (Learmonth et al., 2017). Additionally, age-related weakening of interhemispheric inhibition may release the left hemisphere from right-hemisphere suppression, further contributing to the rightward attentional shift. An alternative account, the HAROLD model (Cabeza, 2002), proposes that aging reduces hemispheric specialization via compensatory bilateral recruitment. However, as Benwell et al. note, this model predicts attenuated bias (toward symmetric processing) rather than the systematic rightward bias observed in their study and ours (Benwell et al., 2014). The pattern of results across studies thus favors the disproportionate right-hemisphere decline account over a simple compensation model.

A key question raised by our findings is whether the age-related directional asymmetry in RFT performance reflects changes in early sensory processing or in higher-level cognitive mechanisms. Several observations favor a cognitive interpretation. The asymmetry emerged specifically under conditions of reference-frame conflict, in which visual and vestibular cues must be actively integrated and weighted, rather than under conditions dominated by passive sensory registration. Moreover, the clockwise-specific nature of the effect cannot be readily explained by general sensory degradation, which would be expected to impair performance symmetrically across tilt directions. At the population level, vestibular dysfunction in older adults is characterized by progressive, bilateral, and partial loss rather than directional impairment (Agrawal et al., 2020). Notably, overall RFT error magnitude did not correlate significantly with the asymmetry index, suggesting that directional asymmetry cannot be reduced to general performance differences. Finally, the asymmetry is consistent with our broader finding of reduced strategic flexibility in older adults, suggesting impaired adaptive strategy selection rather than diminished sensory precision.

This interpretation is supported by recent evidence that directional biases in spatial perception can arise from cognitive rather than sensory processes. Jiang et al. (2025) showed that a preference for clockwise over counterclockwise rotation emerged only in tasks requiring cognitive interpretation of ambiguous input and was absent in paradigms tapping early sensory processing. Although their study focused on motion perception rather than static orientation, the broader principle applies: when sensory information is ambiguous, higher-level cognitive mechanisms play a decisive role. The intermediate frame tilts that revealed the strongest age-related asymmetry in our study represent precisely such conditions of maximal ambiguity, where visual and gravitational reference frames conflict and veridical vertical must be extracted through active cue integration.

The commonly reported sex difference in RFT performance—typically characterized by greater field dependence in women than in men (Witkin et al., 1977; Abdul Razzak et al., 2015; Willey and Liu, 2022)—was not observed in either of our age groups. This null effect likely reflects the educational characteristics of our sample. Both young and older participants had comparable higher-education backgrounds, with the young cohort recruited from a technical university. Previous research has demonstrated that individuals pursuing technical and scientific fields tend to be more field-independent than the general population (Barrett and Thornton, 1967; Witkin et al., 1977; Lusk and Wright, 1981), and this selection and/or training effect may attenuate or eliminate the typical sex difference. Importantly, the absence of sex effects in both age groups, combined with their matched educational profiles, suggests that the observed age-related directional asymmetry in RFT performance cannot be attributed to demographic confounds and likely reflects genuine changes in spatial orientation processing with aging. While we did not design this study to examine sex effects specifically, our findings suggest that the commonly reported sex difference in field dependence may be moderated by educational background—a possibility that has received limited systematic attention. Future studies explicitly comparing RFT performance across educational profiles could clarify whether the well-established sex effect reflects intrinsic biological differences or sampling and educational biases in the existing literature.

Several limitations of the present study should be acknowledged. First, the cross-sectional design limits causal inference regarding age-related change; longitudinal data are needed to disentangle true aging effects from generational differences. Second, because each participant completed only one trial per tilt angle, test–retest reliability could not be assessed. Third, trial completion time was not recorded, precluding analysis of potential speed–accuracy tradeoffs or age-related differences in response latency; future studies should incorporate timing measures to examine whether older adults' increased errors reflect deliberative slowing or other temporal dynamics.

Fourth, although all participants reported normal or corrected-to-normal vision, visual acuity was not formally assessed. However, the RFT stimuli (rod and frame) subtended large visual angles and did not require fine spatial discrimination, making it unlikely that subtle visual deficits influenced performance substantially. Fifth, vestibular function was not directly assessed using clinical measures such as the subjective visual vertical test, caloric testing, or video head impulse testing. Although bilateral vestibular decline cannot account for the directional asymmetry observed here (as argued above), individual differences in vestibular function may have contributed to overall error variance. Future studies combining the RFT with standardized vestibular assessment could clarify the relative contributions of peripheral vestibular function and central attentional mechanisms to age-related changes in verticality perception.

Sixth, symptoms of cybersickness and visual fatigue were not systematically assessed using standardized instruments such as the Simulator Sickness Questionnaire. This is relevant given recent evidence linking rod-and-frame test performance to susceptibility to cybersickness in virtual environments (Maneuvrier, 2023), as well as reports of age-related differences in vulnerability to VR-induced sickness (Rebenitsch and Owen, 2016). In the present study, no participants reported overt symptoms or withdrew due to discomfort, and the use of a static VR environment without locomotion is generally considered to pose a relatively low risk for cybersickness (Rebenitsch and Owen, 2016). Nevertheless, the presence of subclinical symptoms cannot be excluded, and future studies should incorporate standardized assessments to rule out potential VR-related confounds.

Finally, our older adult cohort was relatively healthy and cognitively intact (mean SMMSE = 28 ± 2). Asymmetry effects may be more pronounced in frailer or cognitively impaired populations, warranting investigation across the broader aging spectrum.

In conclusion, our findings demonstrate that aging affects RFT performance not merely through increased error magnitude but through a direction-specific vulnerability that emerges prominently in the seventh decade of life. This clockwise-specific asymmetry cannot be explained by bilateral vestibular decline or general sensory degradation and instead implicates higher-level cognitive mechanisms involved in resolving reference-frame conflict. The convergence of our results with evidence from rotation perception paradigms, landmark judgments, and studies of spatial attention suggests that directional asymmetry may represent a behavioral signature of age-related decline in right-hemisphere spatial processing. More broadly, our findings highlight that perceptual tasks involving ambiguous or conflicting sensory information provide a particularly sensitive window into the cognitive—rather than sensory—changes that accompany healthy aging.

## Data Availability

The datasets presented in this study can be found in online repositories. The names of the repository/repositories and accession number(s) can be found below: https://data.mendeley.com/datasets/hmpys9rr6s/1.
