# Supplementary material for: Age-related directional asymmetry in the rod-and-frame test
Source: Front Aging Neurosci. 2026 Mar 5;18:1729404. doi: 10.3389/fnagi.2026.1729404 (PMC12999833; doi:10.3389/fnagi.2026.1729404)
Supplement: Supplementary file 1 [file Data_Sheet_1.pdf]

Supplementary Material

1 FIGURES

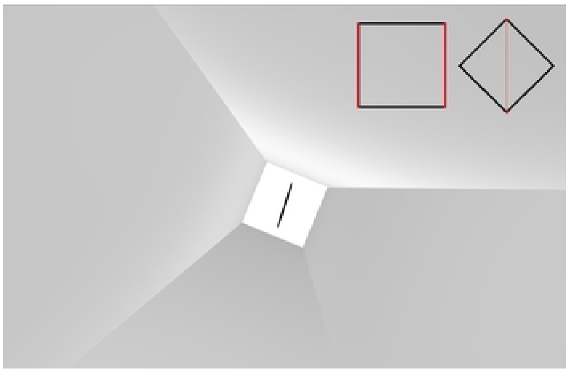

Figure S1. Virtual reality testing room (scene) used in the rod and frame experiment. A subject views a rod of length 1.3 m from a distance of 5 m within a cuboid room with width and height equal to 1.8 m. The room is illuminated by a spotlight positioned behind the subject. The subject determines the subjective visual vertical for 19 randomly presented frame tilt angles ranging from $-45°$ to $45°$ in steps of $5°$. In the upper right corner, the visual cues (attractors) used to infer verticality are shown: the edges of the frame (primary attractor) and one of its diagonals (secondary attractor). Note that this image does not reflect the visual experience of the subjects during the test, as it corresponds to a single-eye perspective.

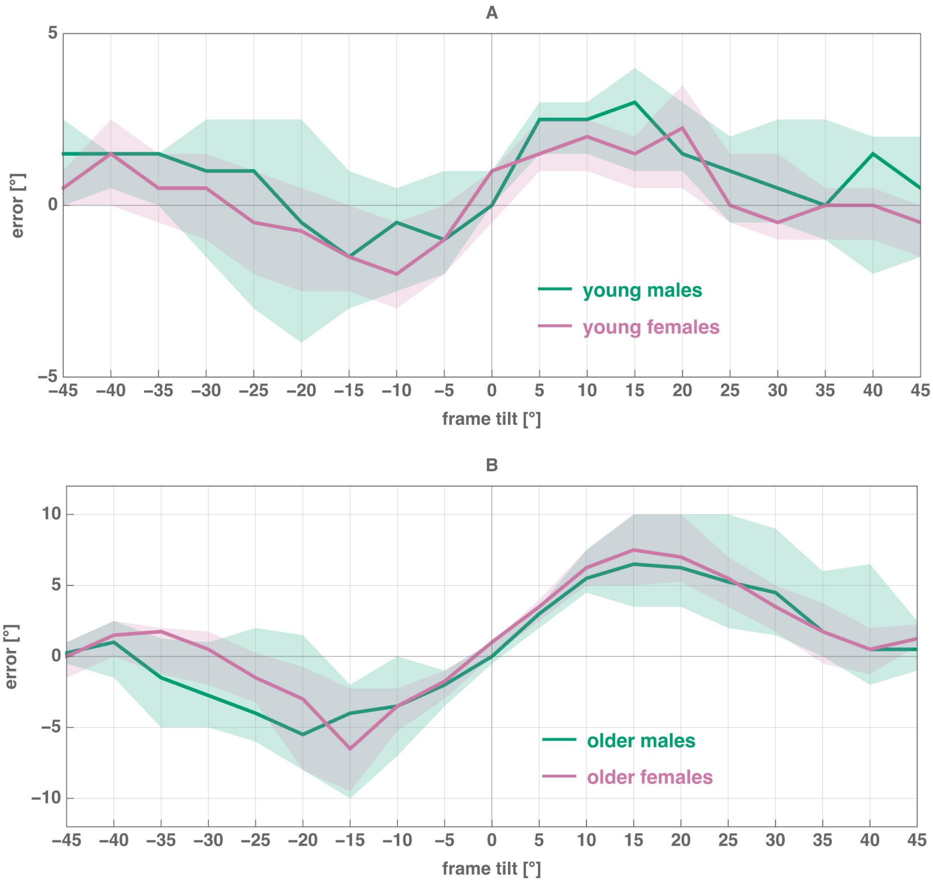

Figure S2. Median error $e(\theta)$ in determining the vertical is plotted as a function of frame tilt θ: (A) young, (B) older adults. In both subplots, data are plotted separately for both sexes. Shading indicates the 95% confidence interval, estimated using the bootstrap method.

Table S1. Mean ($M \pm SD$) and median [IQR] RFT alignment error as a function of frame tilt θ, reported separately for young males ($n = 17$) and females ($n = 22$). The p-values correspond to between-group comparisons between males and females at each tilt angle using a nonparametric Wilcoxon rank-sum (Mann–Whitney U) test and are uncorrected for multiple comparisons.

θ (°)	$M \pm SD$ (M)	$Mdn\,[IQR]$ (M)	$M \pm SD$ (F)	$Mdn\,[IQR]$ (F)	p-value
−45	1.03 ± 1.80	1.50 [0.00, 2.50]	0.84 ± 1.41	0.50 [0.00, 1.37]	0.52
−40	1.41 ± 1.41	1.50 [0.50, 1.50]	1.14 ± 1.85	1.50 [0.00, 2.50]	0.79
−35	1.15 ± 1.80	1.50 [0.00, 1.50]	0.14 ± 2.07	0.50 [−1.25, 1.50]	0.15
−30	0.56 ± 2.62	1.00 [−1.50, 2.50]	−0.25 ± 2.97	0.50 [−2.13, 1.88]	0.44
−25	0.09 ± 3.34	1.00 [−3.00, 2.50]	−0.73 ± 2.35	−0.50 [−2.38, 1.38]	0.27
−20	−1.18 ± 3.35	−0.50 [−4.00, 2.50]	−1.16 ± 2.42	−0.75 [−2.88, 0.88]	0.81
−15	−1.32 ± 2.97	−1.50 [−3.00, 1.00]	−1.73 ± 2.50	−1.50 [−2.88, 0.75]	0.55
−10	−1.00 ± 2.68	−0.50 [−2.50, 0.50]	−1.68 ± 1.84	−2.00 [−3.00, −0.50]	0.27
−5	−0.71 ± 1.98	−1.00 [−2.00, 1.00]	−1.27 ± 2.03	−1.00 [−3.12, 0.38]	0.39
0	0.56 ± 1.55	0.00 [0.00, 1.00]	0.23 ± 1.27	1.00 [−0.50, 1.00]	0.64
5	2.29 ± 1.49	2.50 [1.50, 3.00]	1.75 ± 1.62	1.50 [1.00, 2.50]	0.29
10	1.85 ± 2.80	2.50 [1.50, 3.00]	1.73 ± 1.71	2.00 [1.00, 2.50]	0.42
15	2.59 ± 1.95	3.00 [1.00, 4.00]	1.20 ± 1.91	1.50 [0.50, 2.00]	**0.03**
20	1.88 ± 1.99	1.50 [1.00, 3.00]	1.84 ± 2.33	2.25 [0.50, 3.50]	0.62
25	0.71 ± 2.35	1.00 [−0.50, 2.00]	0.18 ± 2.19	0.00 [−0.50, 1.88]	0.33
30	0.76 ± 2.21	0.50 [−0.50, 2.50]	−0.05 ± 2.46	−0.50 [−1.37, 1.50]	0.24
35	0.32 ± 2.58	0.00 [−1.00, 2.50]	−0.11 ± 1.89	0.00 [−1.00, 0.50]	0.67
40	0.24 ± 2.41	1.50 [−2.00, 2.00]	−0.18 ± 1.80	0.00 [−1.00, 0.50]	0.40
45	0.18 ± 2.17	0.50 [−1.50, 2.00]	−0.50 ± 2.02	−0.50 [−1.50, 0.38]	0.36

Table S2. Mean ($M \pm SD$) and median [IQR] RFT alignment error as a function of frame tilt θ, reported separately for older males ($n = 22$) and females ($n = 28$). The p-values correspond to between-group comparisons between males and females at each tilt angle using a nonparametric Wilcoxon rank-sum (Mann–Whitney U) test and are uncorrected for multiple comparisons.

θ (°)	$M \pm SD$ (M)	$Mdn\,[IQR]$ (M)	$M \pm SD$ (F)	$Mdn\,[IQR]$ (F)	p-value
−45	1.34 ± 7.63	0.25 [−0.50, 1.37]	−0.38 ± 2.50	0.00 [−2.50, 1.50]	0.61
−40	−0.91 ± 9.42	1.00 [−1.88, 2.88]	1.30 ± 3.06	1.50 [−0.50, 2.50]	0.37
−35	−2.59 ± 8.48	−1.50 [−5.38, 1.38]	0.29 ± 4.84	1.75 [−2.63, 3.00]	0.12
−30	−2.52 ± 7.12	−2.75 [−5.38, 1.00]	−1.68 ± 8.44	0.50 [−2.25, 2.12]	0.13
−25	−4.27 ± 6.73	−4.00 [−6.75, 2.00]	−3.07 ± 6.72	−1.50 [−4.13, 1.62]	0.36
−20	−4.98 ± 7.73	−5.50 [−8.75, 2.25]	−5.39 ± 6.82	−3.00 [−9.00, −0.50]	0.76
−15	−4.98 ± 6.08	−4.00 [−10.4, −0.50]	−6.52 ± 5.86	−6.50 [−11.0, −1.00]	0.47
−10	−3.18 ± 4.95	−3.50 [−7.38, 0.37]	−3.64 ± 4.18	−3.50 [−6.50, −1.38]	0.77
−5	−1.09 ± 4.50	−2.00 [−3.50, 0.12]	−1.95 ± 2.26	−1.75 [−3.50, −0.38]	1.00
0	0.27 ± 1.48	0.00 [−0.50, 1.00]	0.66 ± 1.26	1.00 [0.00, 1.12]	0.25
5	1.61 ± 5.54	3.00 [2.00, 3.50]	3.13 ± 1.59	3.50 [2.38, 4.12]	0.31
10	4.86 ± 5.37	5.50 [4.50, 7.88]	6.09 ± 2.59	6.25 [4.88, 8.00]	0.55
15	6.39 ± 5.99	6.50 [3.50, 10.8]	6.98 ± 4.63	7.50 [4.38, 10.0]	0.71
20	6.68 ± 7.12	6.25 [1.62, 10.4]	7.89 ± 5.65	7.00 [5.00, 11.5]	0.52
25	6.68 ± 7.50	5.25 [0.88, 11.5]	5.77 ± 5.75	5.50 [3.00, 8.00]	0.81
30	5.77 ± 7.91	4.50 [0.75, 9.38]	3.63 ± 7.79	3.50 [−0.12, 5.88]	0.33
35	5.52 ± 9.60	1.75 [0.00, 7.50]	2.07 ± 5.51	1.75 [−1.62, 4.25]	0.38
40	4.20 ± 9.87	0.50 [−2.37, 8.38]	1.27 ± 5.76	0.50 [−2.00, 2.50]	0.56
45	1.16 ± 9.11	0.50 [−1.75, 2.88]	2.54 ± 6.15	1.25 [0.38, 3.00]	0.41

Table S3. Asymmetry index α_e by sex in young ($n_M = 17$, $n_F = 22$) and older adults ($n_M = 22$, $n_F = 28$). Values are mean ± SD. The p-values correspond to between-sex comparisons within each cohort using independent-samples t-tests.

Group	$M \pm SD$ (M)	$M \pm SD$ (F)	p-value
Young	0.035 ± 0.243	-0.069 ± 0.219	0.1609
Seniors	0.088 ± 0.262	0.156 ± 0.261	0.5001
